# Brucella prostatitis presenting with prostatic abscess in a herdsman: a case report and literature review

**DOI:** 10.1097/RC9.0000000000000310

**Published:** 2026-03-04

**Authors:** Noor AlBuloushi, Mohammed Zahir, Mohammad AlFahad, Abdullah Al Enezi

**Affiliations:** a Urology Department, Jaber Al Ahmed Al Jaber Al Sabah Hospital, Kuwait; b Kuwait Ministry of Health, Kuwait; c Kuwait Urology Board, Kuwait Institute for Medical Specialization, Kuwait

**Keywords:** brucella prostatitis, case report, infectious diseases, mimic malignancy, prostate cancer, Prostatitis, zoonotic infectious disease

## Abstract

**Introduction:**

Brucellosis is a zoonotic infection caused by Brucella species, primarily transmitted through direct contact with infected animals or consumption of unpasteurized dairy products. Genitourinary involvement is uncommon, with prostatic infection being rare. We present a case of Brucella prostatitis with prostatic abscess in a herdsman, initially mimicking malignancy on imaging making it a challenging clinical entity.

**Case Presentation:**

We report a case of a 43-year-old male and highlighting the challenges in diagnosis and management of this uncommon manifestation of Brucellosis. He was a herder from an endemic area that presented with recurrent lower urinary tract symptoms over several visits. In his last visit he presented with a high-grade fever and dysuria. Although he initially denied risky exposures, he later admitted to consuming unpasteurized dairy products. Physical examination revealed a mildly tender prostate on digital rectal examination. Laboratory studies showed elevated inflammatory markers, and blood cultures grew Brucella melitensis. Transrectal ultrasound demonstrated a hypoechoic lesion consistent with a prostatic abscess. The patient was treated with doxycycline and rifampin for 8 weeks, resulting in full clinical recovery without relapse at follow-up.

**Discussion:**

Brucella prostatitis is rare, often misdiagnosed due to nonspecific symptoms and radiologic overlap with prostate cancer. Few cases are reported in the literature, most in occupationally exposed individuals. Our case emphasizes the need to consider Brucella in endemic areas, particularly in patients with unexplained prostatic abscess.

**Conclusion:**

The case report underscores the importance of considering brucellosis as a differential diagnosis, particularly in patients with occupations that have close encounters with animals. Early recognition of Brucella prostatitis allows prompt initiation of targeted antibiotic therapy, preventing complications and unnecessary surgical intervention.

## Introduction

Brucellosis is a zoonotic infectious disease caused by small, aerobic, intracellular coccobacilli of the genus Brucella^[^1^]^. It is transmitted to humans primarily through direct contact with infected animals or consumption of unpasteurized animal products^[^1^]^. While osteoarticular involvement is the most frequent focal complication, the genitourinary system is affected in 2–20% of cases, most commonly as epididymo-orchitis^[^2^]^. Prostatic involvement is rare and may present diagnostic challenges, due to it closely mimicking other common bacterial prostatitis or even prostate cancer. Routine urine cultures fail to detect Brucella, and specific serological or culture tests are usually needed, however not requested unless there is a high index of suspicion. Furthermore, imaging findings are not pathognomonic nor specific which further contributes to it being underdiagnosed or delayed in treatment^[^3^]^. This case report has been reported in line with SCARE 2025 criteria^[^4^]^.

**Figure 1.**
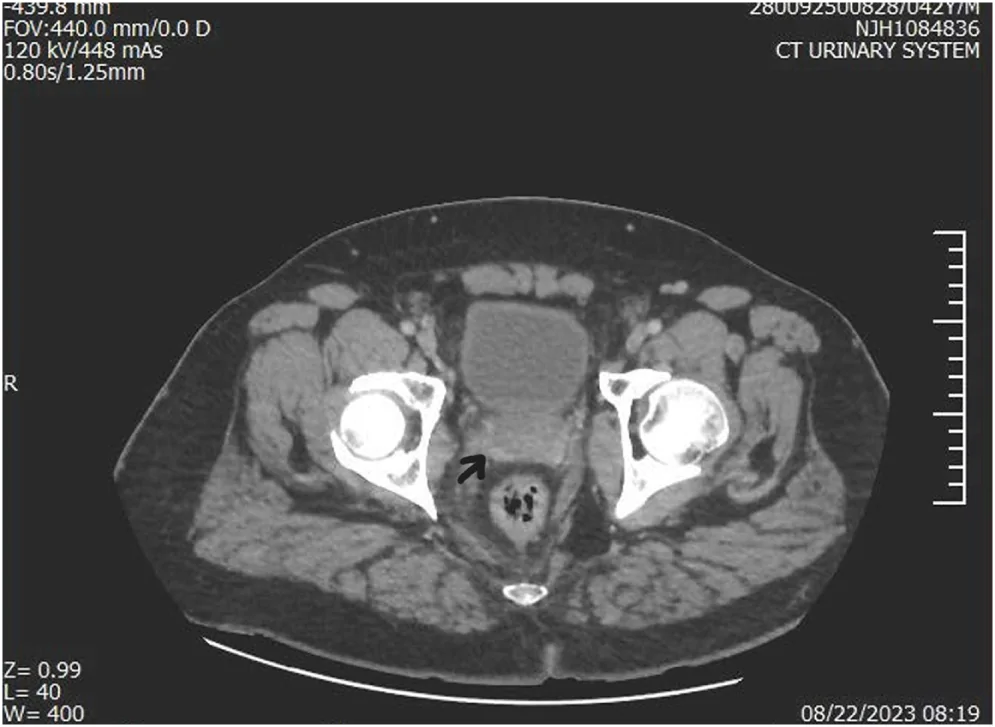

HIGHLIGHTS

- brucella prostatitis is a rare genitourinary manifestation of brucellosis.
- occupational exposure and unpasteurized dairy consumption are key risk factors.
- presentation may mimic malignancy on imaging, leading to misdiagnosis.
- early diagnosis and prolonged dual antibiotic therapy are crucial for cure.

## Case presentation

A 43-year-old male herder from an endemic region presented with recurrent lower urinary tract symptoms (LUTS), including frequency, urgency, and dysuria, over multiple emergency department visits. He subsequently developed high-grade fever and worsening dysuria. Initially denying risky exposures, he later admitted to regular consumption of unpasteurized dairy products, believing in their health benefits.

On examination, the patient was febrile and hemodynamically stable. Laboratory investigations showed elevated white blood cell count of 21.7 K/μl and CRP of 77.3 mg/liter, with normal PSA. Blood cultures were positive for Brucella melitensis as well as serological testing for antibodies using Brucella Agglutination test, called Rose Bengal test were positive. Transrectal ultrasound revealed a hypoechoic lesion suggestive of a prostatic abscess.

The patient was admitted for monitoring and started on piperacillin and tazobactam 4.5 g three times a day, and cefotaxime 1 gm every 8 hours for the first week. He was initiated on doxycycline (100 mg twice daily) and rifampin (600 mg once daily) for 8 weeks on discharge. Serial imaging demonstrated significant reduction in abscess size and repeat blood cultures were negative at 6 weeks. At follow-up, the patient reported complete symptom resolution, with no relapse.

## Discussion

Brucella prostatitis is a rare manifestation of brucellosis. The condition may mimic other prostatic pathologies, including bacterial prostatitis and malignancy. In our case, the coexistence of prostatic abscess and occupational exposure history pointed toward a zoonotic etiology.

Prostatic involvement by Brucella is often underdiagnosed due to nonspecific symptoms and low clinical suspicion as well as accounting for 2.7% of all cases of prostatitis^[^5^]^. Its seasonal incidence is between May to August with a peak in July.^[^6^]^ Brucella prostatitis has no specific symptoms to distinguish it from other urinary tract infections making is extremely difficult for diagnose.

Diagnosis is confirmed via culture or serology^[^7^]^. Imaging aids in identifying abscess formation. The treatment is generally with antibiotics such as doxycycline and rifampin as recommended by WHO^[^8^]^. It does have a relapse rate of about 4.6% to 24.3%^[^9^]^. This makes the follow up and monitoring essential with clinical evaluation and laboratory tests after 6 weeks of treatment. Nevertheless, if left untreated it may progress to chronic prostatitis or even abscess formation. It’s crucial for healthcare professionals to help spread awareness, especially in endemic areas on prevention and precautions, including avoid consumption of unpasteurized dairy products, good hygiene especially who are in close contact with animals.

Table 1 summarizes similar cases reported in the literature^[^10–12^]^.

**Table 1:** Reported cases of Brucella prostatitis.

Author/Year	Country	Patient Age	Presentation	Outcome
Aksoy *et al.*, 2009	Turkey	55	Prostatitis mimicking carcinoma	Recovered with antibiotics
Alenazi *et al.*, 2018	Saudi Arabia	48	Prostatic abscess	Recovered with antibiotics
Ben Arab *et al.*, 2008	Tunisia	52	Prostatic abscess	Recovered with antibiotics
Present case	Endemic region	43	Prostatic abscess, LUTS, fever	Recovered with antibiotics

## Conclusion

Brucella prostatitis, though rare, should be considered in patients from endemic areas presenting with prostatic abscess, especially with relevant occupational or dietary exposures. Early diagnosis and prolonged dual antibiotic therapy can result in excellent outcomes, avoiding unnecessary surgical intervention.

## Data Availability

The data that support the findings of this study are available from the corresponding author upon reasonable request.

## Research registration unique identifying number (UIN)

1. Researchregistry. 2. researchregistry10233, 3. https://www.researchregistry.com/register-now#home/registrationdetails/662d2aa2ad1e3b0027a50daa/,
